# SSRI Facilitated Crack Dancing

**DOI:** 10.1155/2017/4318450

**Published:** 2017-04-11

**Authors:** Ravi Doobay, Lili Sun, Amish Shah, Pardeep Masuta, Zachary Shepherd

**Affiliations:** Internal Medicine, SUNY Upstate Medical University, 750 E Adams St, Syracuse, NY 13210, USA

## Abstract

Choreoathetoid movement secondary to cocaine use is a well-documented phenomenon better known as “crack dancing.” It consists of uncontrolled writhing movements secondary to excess dopamine from cocaine use. We present a 32-year-old male who had been using cocaine for many years and was recently started on paroxetine, a selective serotonin reuptake inhibitor (SSRI) for worsening depression four weeks before presentation. He had been doing cocaine every 2 weeks for the last three years and had never “crack danced” before this episode. The authors have conducted a thorough literature review and cited studies that suggest “crack dancing” is associated with excess dopamine. There has never been a documented case report of an SSRI being linked with “crack dancing.” The authors propose that the excess dopaminergic effect of the SSRI lowered the dopamine threshold for “crack dancing.” There is a communication with the Raphe Nucleus and the Substantia Nigra, which explains how the SSRI increases dopamine levels. This is the first documented case of an SSRI facilitating the “crack dance.”

## 1. Introduction

Choreoathetoid movement secondary to cocaine use also known as the “crack dance” is a documented phenomenon that is surprisingly more common in the younger patient population [1]. Choreoathetosis literally means the occurrence of involuntary movements with twisting and writhing [1]. We present a first-time occurrence of the “crack dance” one month after beginning paroxetine, a selective serotonin reuptake inhibitor (SSRI) in the context of cocaine abuse. We propose that the excess dopaminergic effect of the SSRI lowered the threshold for the choreoathetoid movements. This is the first documented case report of the “crack dance” being associated with an SSRI.

## 2. Case Presentation

We present a 32-year-old male with a past medical history of hypertension, major depression, and cocaine abuse who presented with involuntary head, jaw, tongue, facial, shoulder, trunk, and leg movements; they were rhythmic and choreiform. Voluntary movements would worsen the choreoathetosis. He did cocaine three days before presentation. He initially complained of fevers, chills, and diaphoresis two days before presentation. He then noticed abnormal movements of his head, which then progressed into uncontrolled movements of his arms, shoulder, trunk, and legs. He denied chest pain, dyspnea, nausea, vomiting, diarrhea, dysuria, or leg swelling. He had been recently put on paroxetine by his primary care physician four weeks ago for major depression. He claimed to have not done any other recreational drugs, which was supported by a urine toxicology screen. He presented with a temperature of 37.1°C, Pulse of 90, RR of 20, BP of 165/101 mmHg, and saturating 95% on room air. A comprehensive metabolic panel and thyroid stimulating hormone were within normal limits. There were no pertinent positives on physical exam other than the uncontrolled movements; he remained alert and oriented at all times. Apart from the choreoathetosis the neurological examination was within normal limits. There was no parkinsonian or dystonic features to suggest an alternate diagnosis. He received diphenhydramine and benztropine from the Emergency Department physicians without relief. Toxicology was consulted and concluded these were indeed choreoathetoid movements secondary to cocaine use. There was no head imaging ordered as the diagnosis was established. He was admitted to Medicine and symptoms resolved with IV Lorazepam and intravenous fluids in 48 hours. During these 48 hours the uncontrolled movements were continuous.

## 3. Discussion

The differential diagnoses for acute adult-onset nonhereditary choreas are autoimmune (systemic lupus erythematosus, polyarteritis nodosa, Behcet's disease, Sjögren's syndrome, Sydenham's chorea, and antiphospholipid syndrome), metabolic (hyponatremia, hypoglycemia, hypocalcemia, and hyperthyroidism), infectious (neurosyphilis, lyme disease, and AIDS), drugs (neuroleptics, amphetamines, lithium, and digoxin), and malignancy with basal ganglia involvement [2]. The symptoms resolved with IV benzodiazepines, fluids, and symptomatic management which suggests a cocaine induced choreoathetosis, especially with the patient's history. Cocaine is known to have stimulating effects on behavior. It acts by blocking the reuptake of certain catecholamines such as dopamine, norepinephrine, and serotonin by blocking transporter receptors [2]. More specifically, cocaine acts on dopamine transporter receptors thereby blocking dopamine reuptake which results in a rapid increase in dopamine availability at the synaptic cleft [2]. Studies have shown that chronic cocaine use may lead to a compensatory response to overcome the dopaminergic overstimulation [2]. Cocaine also decreases tyrosine hydroxylase activity, an enzyme used in production of dopamine, and postsynaptic dopamine receptor sites which would overall decrease the availability of dopamine and its effect. However, failure to engage in this mechanism and downregulate dopamine results in the occurrence of choreoathetoid movements [2].

While SSRIs by definition inhibit serotonin reuptake from brain synapse, they also have an affinity for norepinephrine and dopamine receptors. In our patient, it is likely that with such chronic history of cocaine abuse, his recent regular usage of paroxetine synergistically added to the effect of increasing dopamine at the synapse thereby lowering the threshold for the development of choreoathetosis.

There are compelling reasons why excess dopamine in the basal ganglia causes chorea. Sutamtewagul et al. (2014) showed a similar presentation in a 37-year-old male after taking 3 consecutive doses of “bath salts,” a well-known street drug, intravenously [3]. The active compounds in bath salts are 3,4-methylene-dioxypyrovalerone or 4-methylmethcathinone which inhibit dopamine reuptake [3]. Stork and Cantor (1997) documented choreoathetosis secondary to Pemoline use [4]. Pemoline is an oxazolidine derivative that is a dopaminergic agonist [4]. It is used to treat attention deficit disorder. Two three-year-old males ingested multiple Pemoline tablets and were found to be in choreoathetosis [4]. They were treated with IV benzodiazepines and the symptoms resolved in 48 hours [4]. There was no history of movement disorder or family history of movement disorders in these patients [4]. Also choreoathetoid movements induced by levadopa is very well documented in Parkinson patients [5]. We suspect in this particular patient that the SSRI caused release of serotonin in the Raphe Nucleus which has communication with the Substantia Nigra [6] leading to increased dopamine resulting in uncontrolled movements better known as “crack dancing” [6].

In conclusion choreoathetosis secondary to cocaine use also known as the “crack dance” is secondary to an excess dopaminergic effect. We present a first-time “crack dance” facilitated by an SSRI in a 32-year-old male who has done cocaine every 2 weeks for the last three years. We propose that the SSRI started by his PCP four weeks ago increased the dopamine activity above the threshold for choreoathetosis. This is the first case report linking SSRI use to “crack dancing.” There are two reasons why the authors believe the SSRI induced the choreoathetosis: it was four weeks after the initiation of the SSRI which is when it starts to take effect; also the patient had done cocaine for many years and this was a first-time occurrence of choreoathetosis. This case also demonstrates the potential risk of SSRI use in chronic cocaine users predisposing them to involuntary movements. There are only a handful case reports of choreoathetosis secondary to cocaine use and there has never been a documented association with SSRIs. We present the first SSRI facilitated “crack dance.”
